# Algorithms of Electrocardiographic Changes for Quantitative and Localization Analysis of Thrombus Burden in Patients with Acute Pulmonary Thromboembolism

**DOI:** 10.31083/j.rcm2410281

**Published:** 2023-10-07

**Authors:** Fan Wang, Lan Wang, ChunXi Yan, Xiaoxin Chang, Huaping Wang, Kaiyuan Zhu, Yawei Xu, Dachun Xu

**Affiliations:** ^1^Department of Cardiology, Shanghai Tenth People's Hospital, Tongji University School of Medicine, 200072 Shanghai, China; ^2^Department of Pulmonary Circulation, Shanghai Pulmonary Hospital, Tongji University School of Medicine, 201209 Shanghai, China; ^3^Department of Cardiology, Qidong People's Hospital Affiliated to Nantong University, 226200 Nantong, Jiangsu, China; ^4^Department of Hematology, Shanghai Tenth People's Hospital, Tongji University School of Medicine, 200072 Shanghai, China

**Keywords:** acute pulmonary thromboembolism, electrocardiographic changes, thrombus burden, risk model

## Abstract

**Background:**

Various electrocardiographic (ECG) abnormalities are associated with the severity of pulmonary thromboembolism (PTE). The utility of evaluating the clot burden of PTE based on ECG findings alone has yet to be thoroughly investigated in Chinese patients. The aim of this study was therefore to use ECG signs to establish novel models for quantitative and localization analysis of clot burden in patients with acute PTE.

**Methods:**

Acute PTE patients from three centers were enrolled between 2015 and 2019 in a retrospective cohort study (NCT03802929). We analyzed the 12-lead ECGs at admission and studied computed tomography pulmonary angiography (CTPA) features to obtain the Qanadli score of clot burden and location of thrombus. Novel risk prediction models were developed and validated using derivation and external validation cohorts, respectively.

**Results:**

A total of 341 acute PTE patients were screened, of whom 246 (72.1%) were from Shanghai Tenth People’s Hospital, 71 (20.8%) were from Shanghai Pulmonary Hospital and 24 (7.0%) were from Qidong People’s Hospital. In the derivation cohort, predictors included in the final models were congestive heart failure, chronic obstructive pulmonary disease, hypertension, coronary heart disease, atrial fibrillation and ECG abnormalities. The CHARIS (**C**OPD/CHF/CHD, **H**TN, **A**trial arrhythmias/AF, **R**BBB/RAD, **I**nverted T wave and **S**1Q3T3/ Sinus tachycardia) I model was established for quantitatively assessing Qanadli score. It had moderate discrimination in both the derivation cohort (concordance index (c-index) of 0.720, 95% CI 0.655–0.780) and the validation cohort (c-index of 0.663, 95% CI 0.559–0.757). The CHARIS II model was used to predict the probability of trunk obstruction. It showed similar discrimination in the derivation cohort (c-index of 0.753, 95% CI 0.691–0.811) and in the validation cohort (c-index of 0.741, 95% CI 0.641–0.827). Calibration curves and Hosmer-Lemeshow test confirmed the accuracy of the risk prediction equations in the external validation dataset. Decision curve analysis showed the CHARIS I and CHARIS II algorithms had positive net benefits in both the derivation and validation cohorts.

**Conclusions:**

From quantitative and localization perspectives, the CHARIS algorithms can identify acute PTE patients with heavy thrombus burdens prior to imaging diagnosis.

**Clinical Trial Registration:**

NCT03802929, https://www.clinicaltrials.gov/study/NCT03802929.

## 1. Introduction

Acute pulmonary thromboembolism (APTE) is the third most frequent cause of cardiovascular morbidity and mortality after acute coronary syndrome and stroke. With improvements in diagnostic modalities and physician awareness, the inpatient mortality of APTE in China decreased progressively from 25.1% in 1997 to 8.7% in 2008 [1]. The clinical presentations of APTE varies, ranging from asymptomatic and incidentally discovered emboli, to massive embolism causing sudden death prior to diagnosis. In Europe, 34% of pulmonary thromboembolism (PTE) patients suffer immediate death, 59% remain undiagnosed until death, and only 7% having a definitive diagnosis before death [2]. Hence, timely diagnosis of PTE and screening out massive PTE can be challenging, and the overall mortality from massive pulmonary embolism (PE) remains as high as 52% [3].

Electrocardiographic (ECG) changes are increasingly reported as being useful for assessing the severity of PE, while also showing a strong correlation with hemodynamic collapse of right ventricular dysfunction [4, 5, 6, 7, 8, 9, 10]. So far, there is a notable absence of ECG in the clinical tools available for prognostication of PE in the guidelines. To address this, Daniel *et al*. [11] reported an ECG scoring system in 2001 that could predict the severity of pulmonary artery hypertension (PAH) in PTE and the degree of perfusion defect on ventilation-perfusion lung scanning. However, few Chinese studies have so far evaluated the utility of ECG abnormalities for assessing clot burden, hemodynamic status, right ventricular function, survival rates and management of patients presenting with acute PTE. The initial aim of this study was therefore to develop a revised scoring system that could predict the severity of acute PTE (localization and quantification) on the basis of ECG abnormalities. This should assist clinicians when screening patients who present with massive PTE prior to imaging diagnosis.

## 2. Methods

### 2.1 Study Design and Data Source

This study was registered with the American Clinical Trials Registry Center (https://clinicaltrials.gov; Registration number NCT03802929) and approved by our institutional review board. Informed consent was obtained from all participants or their families.

We conducted a multicenter retrospective cohort study involving consecutive inpatients with confirmed APTE. The three hospitals were the Shanghai Tenth People’s Hospital, the Shanghai Pulmonary Hospital, and the Qidong People’s Hospital. Eligibility for this study required patients to be >18 years of age and have acute PTE confirmed by an intraluminal filling defect on multidetector computed tomography pulmonary angiography (MCTPA). Exclusion criteria were the absence of medical information, pregnancy, or unavailable MCTPA data.

### 2.2 Derivation and External Validation Cohorts

The final selected research subjects were divided into derivation and validation groups according to the different thrombosis centers. The derivation cohort consisted of 246 acute PTE patients hospitalized at Shanghai Tenth People’s Hospital, China, between April 2015 and December 2019. The external validation cohort consisted of 95 acute PTE patients recruited from Shanghai Pulmonary Hospital, China (n = 71) and Qidong People’s Hospital, China (n = 24), also between December 2015 and December 2019.

### 2.3 Electrocardiogram

We retrospectively analyzed 12-conductive ECG performance of all selected patients when acute PTE was confirmed. ECGs were recorded using a standard voltage of 1 mV/10 mm and a paper passing speed of 25 mm/s. ECG changes were independently analyzed by an ECG doctor and a cardiologist. Six common ECG manifestations associated with the prognosis of PTE were evaluated in this study: (1) typical S1Q3T3 signs, (2) sinus tachycardia, (3) complete/incomplete right bundle branch block (RBBB), (4) right axis deviation (RAD), (5) atrial arrhythmias, (6) V1–V3 T wave inversion (TWI) (Table 1).

**Table 1.** **Common ECG findings and their definitions in patients with APTE**.

ECG changes	Definition
∙ typical S1Q3T3 signs	∙ prominent S-wave in lead I and Q/q-wave with T-wave inversion in lead III
∙ sinus tachycardia	∙ heart rate >100 bpm
∙ complete/incomplete RBBB	∙ rsR pattern V1–V3 with or without QRS duration >120 ms
∙ right axis deviation	∙ frontal axis falls at +90°~+180°
∙ atrial arrhythmias	∙ new onset of atrial premature complexes (APCs) or atrial flutter or atrial fibrillation
∙ V1–V3 T wave inversion	∙ Simultaneous T wave inversion in the right precordial leads (V1–V3)

ECG, electrocardiographic; APTE, acute pulmonary thromboembolism; RBBB, right bundle branch block.

### 2.4 Computed Tomography Pulmonary Angiography

**Fig. 1.**
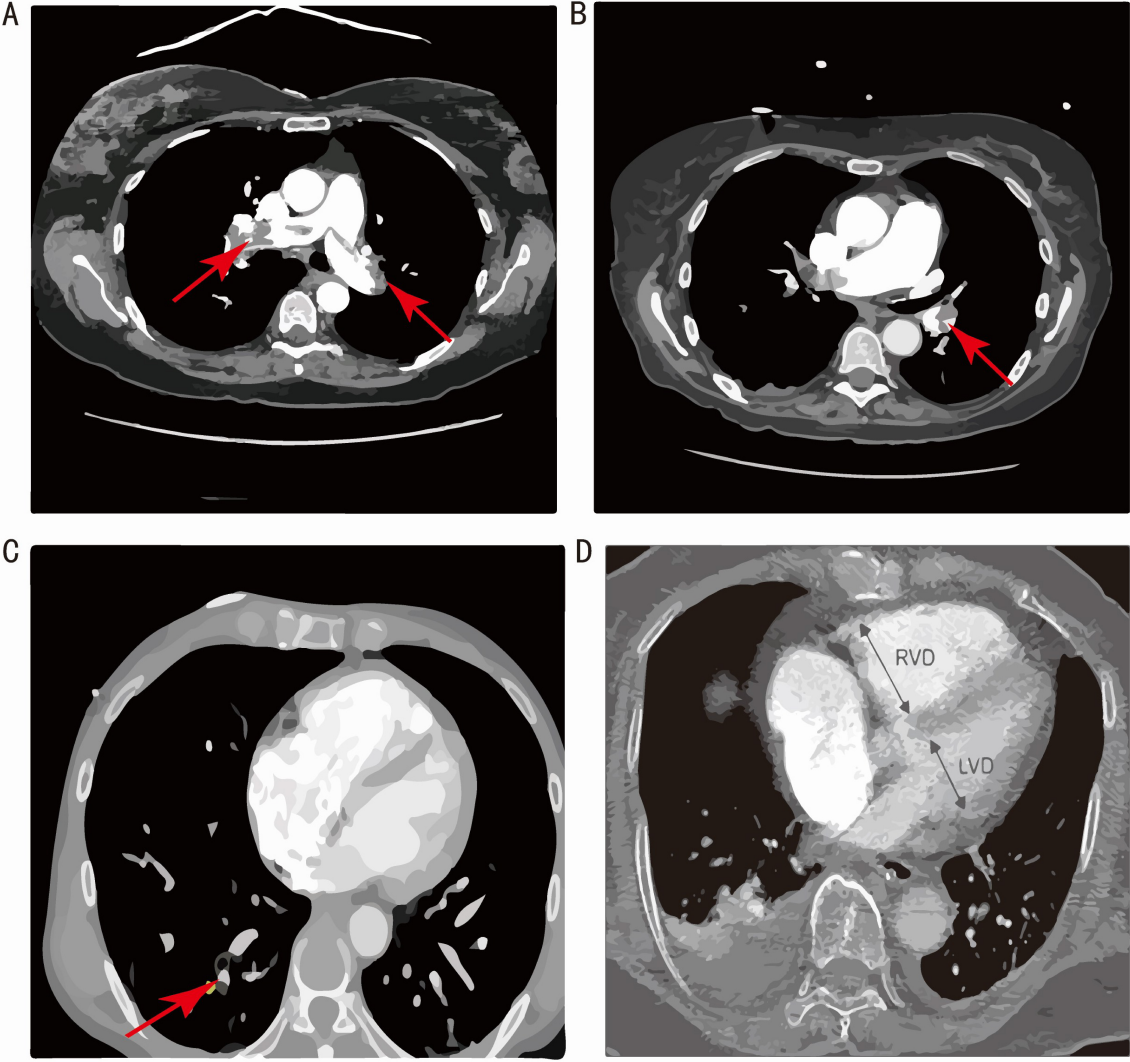
**MCTPA images demonstrating thrombus in different parts of the pulmonary artery (PA).** (A) large saddle embolus at the bifurcation of the main pulmonary artery. (B) Blood clot in the lobar branch of PA. (C) Blood clot in the segmental branch of PA. (D) MCTPA illustration of RV/LV diameter ratio measurement on a four-chamber CT image. RVD, right ventricular dysfunction; LVD, left ventricular dysfunction; MCTPA, multidetector computed tomography pulmonary angiography; RV, right ventricle; LV, left ventricle; CT, computerized tomography.

All selected subjects in this study underwent MCTPA. The examination was performed by a technician accompanied by a clinician, and the report was independently reviewed by at least two radiologists. MCTPA images were used to obtain thrombus positions for locative assessment of clot burden (Fig. 1A–C), while the Qanandli score was calculated for the quantitation of clot burden. The arterial tree of each lung is regarded as having 10 segmental pulmonary arteries (PAs) (three to the upper lobes, two to the middle lobe or lingula, and five to the lower lobes). Qanadli score = ∑(n*d)/40 × 100%, “n” indicates the number of blocked PA segments, “d” indicates the severity of PA blockage, “d = 0” means no defect, “d = 1” means partial occlusion, “d = 2” means complete blockage [12]. MCTPA was used to assess right ventricular dysfunction (RVD), defined as a ratio of right ventricle (RV) to left ventricle (LV) short-axis diameters >0.9 (Fig. 1D).

### 2.5 Selection of Risk Factors for Development of the Model

For each patient, information was extracted on sociodemographic and lifestyle characteristics, pre-existing comorbidities, hematological parameters, ECG performance, and imaging examination results from medical records. ECG changes and comorbidities affecting ECG performance were selected as candidate predictors based on an extensive literature review. These comorbidities included chronic obstructive pulmonary disease (COPD), hypertension (HTN), coronary heart disease (CHD), congestive heart failure (CHF), atrial fibrillation (AF).

### 2.6 Statistical Analysis

All analyses were performed with Stata 15.0 (Statacorp LLC, College Station, TX, USA). Continuous variables with normal distribution were presented as mean ± standard deviation. Numerical variables with skewed distribution were expressed as median and interquartile range. Categorical variables were expressed as frequency and percentages. Comparison of variables between groups was performed using the one-way analysis of variance (ANOVA) for continuous parameters, and the χ^2^ test or Fisher’s exact test for categorical variables as appropriate. In the derivation cohort, a binary logistic regression analysis was performed to establish risk prediction models for quantifying thrombus burden and for evaluating the odds of thrombus in the main PA. Both internal and external validation were performed. Receiver operating characteristic (ROC) curve analysis was used to estimate the discriminatory power of the rules with area under the curve (AUC) (or c-index). Calibration plot was applied to provide insight into the calibrating potential of the new models and the Hosmer-Lemeshow test was used to examine the so-called ‘goodness-of-it’. Decision curve analysis (DCA) was conducted to determine the clinical utility of the new models by evaluating net benefits. For all analysis, a 2-tailed *p* value < 0.05 was used to define statistical significance.

## 3. Results

### 3.1 Study Participants

**Fig. 2.**
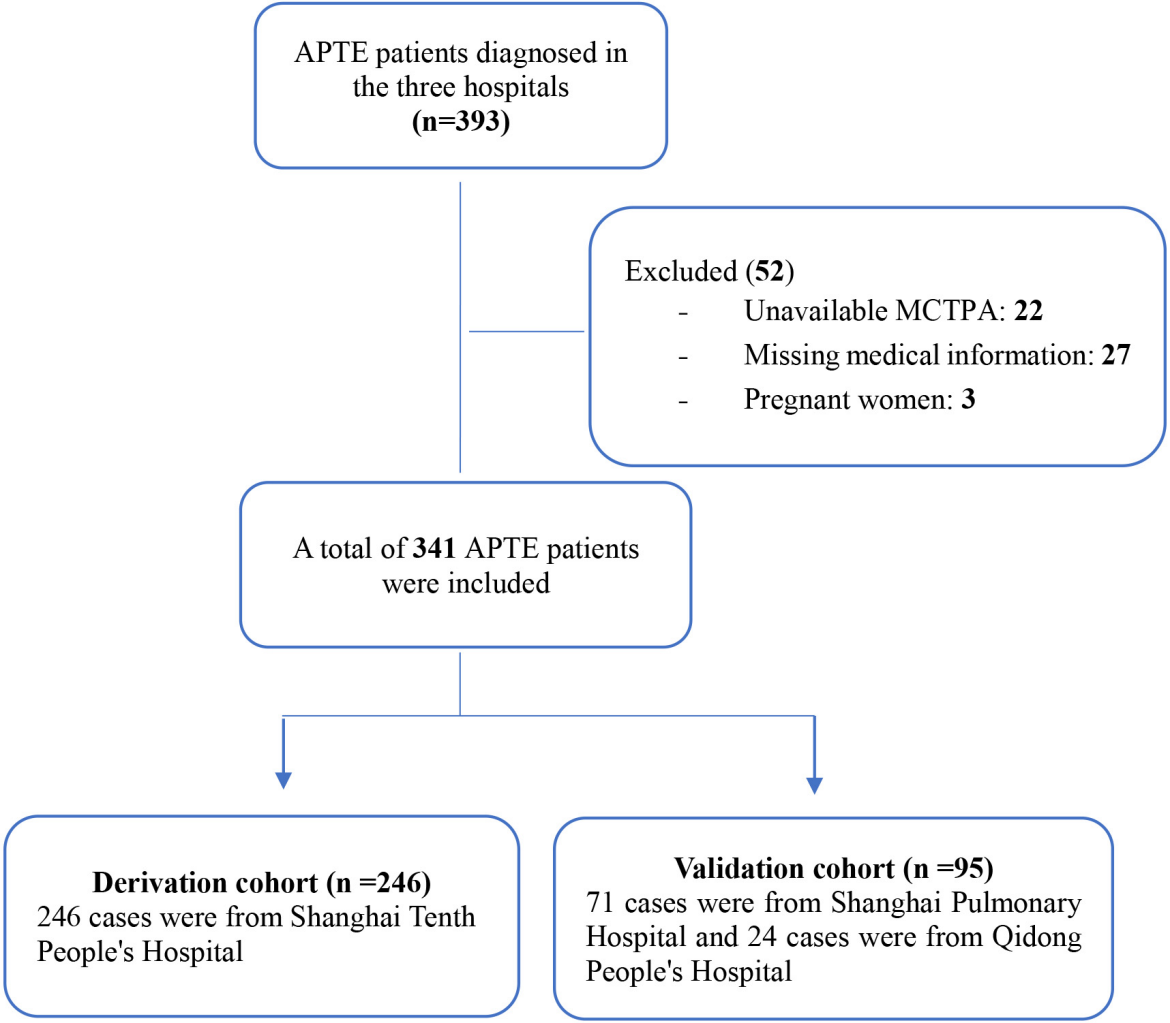
**Patient flow diagram.** APTE, acute pulmonary thromboembolism; MCTPA, multidetector computed tomography pulmonary angiography.

Overall, 393 consecutive patients with acute PTE were screened for eligibility. Of these, 22 (5.6%) patients were excluded because they did not have a technically adequate MCTPA according to the established criteria. Of the remaining 371 patients, 30 were excluded to pregnancy (n = 3, 0.8%) or missing medical information (n = 27, 6.9%), leaving 341 eligible patients enrolled in the study. The subjects were assigned to either the derivation group or validation group according to the hospital at which they were treated (Fig. 2).

### 3.2 General Characteristics of Study Subjects

The general characteristics of the two groups are listed in Table 2. In the derivation group (n = 246), 128 patients (52%) were males and the average age was 72 (±13) years. Of the 246 cases, 55 patients (22.4%) suffered from COPD,143 (58.1%) had HTN, 43 (17.5%) had CHD, 25 (10.2%) had CHF, and 24 (9.8%) had AF. In the validation group, 44 patients (46.3%) were males, and the average age was 61 (±15) years. In this group, 18 patients (18.9%) had COPD, 44 (46.3%) had HTN, 17 (17.9%) had CHD, 13 (13.7%) had CHF and 7 (7.4%) had AF.

**Table 2.** **Baseline Characteristics of Patients in the Derivation and Validation Cohorts**.

Variables		Derivation cohort	Validation cohort
		n = 246	n = 95
Gender (male) (%, n)		52.0 [128]	46.3 [44]
Age (years)		72 ± 13	61 ± 15
BMI (${\mathrm{kg/m}}^{2}$)		24.1 ± 4.1	25.1 ± 3.3
SBP (mmHg)		137 ± 23	134 ± 26
DBP (mmHg)		81 ± 35	78 ± 13
Heart rate (bpm)		90 ± 42	91 ± 20
${\mathrm{SPO}}_{2}$ (%)		95 ± 7	95 ± 3
Comorbidities			
	COPD (yes) (%, n)	22.4 [55]	18.9 [18]
	Hypertension (yes) (%, n)	58.1 [143]	46.3 [44]
	Diabetes mellitus (yes) (%, n)	20.3 [50]	11.6 [11]
	CHD (yes) (%, n)	17.5 [43]	17.9 [17]
	CHF (yes) (%, n)	10.2 [25]	13.7 [13]
	Atrial fibrillation (yes) (%, n)	9.8 [24]	7.4 [7]
	Stroke (yes) (%, n)	18.7 [46]	8.4 [8]
	Cancer (yes) (%, n)	16.7 [41]	13.7 [13]
Laboratory parameters			
	WBCs (×${\mathrm{10}}^{9}$/L)	8.1 ± 3.7	8.5 ± 3.5
	Hemoglobin (g/L)	122 ± 20	130 ± 20
	Platelets (×${\mathrm{10}}^{9}$/L)	214 ± 79	216 ± 76
	ALT (u/L) median (25th–75th percentiles)	25.4 [14.1, 40.0]	27.0 [15.0, 46.0]
	AST (u/L)	26.0 [18.9, 39.6]	25.0 [18.0, 38.0]
	Serum creatine (µmol/L)	80.2 ± 34.7	66.2 ± 23.8
	BUN (mmol/L)	6.9 ± 4.2	6.3 ± 3.3
	UA (µmol/L)	321 ± 113	340 ± 131
	CRP (mg/L)	15.8 [4.9, 54.3]	13.6 [3.2, 23.3]
	cTnT (ng/mL)	0.017 [0.009, 0.042]	0.028 [0.015, 0.054]
	cTnI (ng/mL)		0.006 [0.003, 0.012]
	CK-MB (ng/mL)	1.88 [1.18, 3.67]	3.26 [2.00, 5.30]
	NT-proBNP (pg/mL)	426 [107, 1419]	321 [40, 882]
	D-dimer (mg/L)	5.07 [2.25, 8.27]	1.6 [0.68, 6.2]
Electrocardiogram			
	S1Q3T3 signs (%, n)	6.5 [16]	14.7 [14]
	Sinus tachycardia (%, n)	17.1 [42]	22.1 [21]
	RBBB (%, n)	12.2 [30]	10.5 [10]
	Incomplete RBBB (%, n)	6.5 [16]	8.4 [8]
	Complete RBBB (%, n)	5.7 [14]	2.1 [2]
	Right axis deviation (%, n)	2.4 [6]	5.3 [5]
	Atrial arrhythmias (%, n)	17.1 [42]	14.7 [14]
	V1–V3 TWI (%, n)	12.2 [30]	13.7 [13]
Thrombus position			
	Trunk (%, n)	23.2 [57]	42.1 [40]
	Bilateral trunk (%, n)	11.4 [28]	25.3 [24]
	Left main PA (%, n)	3.3 [8]	2.1 [2]
	Right main PA (%, n)	8.5 [21]	14.7 [14]
	Lobar PA (%, n)	24.0 [59]	26.3 [25]
	Segmental PA (%, n)	48.0 [118]	29.5 [28]
Qanadli score (%)		27.5 ± 25.3	42.0 ± 33.5
	[0–25%] (%, n)	59.8 [147]	47.4 [45]
	[25%–50%] (%, n)	24.4 [60]	18.9 [18]
	[50%–75%] (%, n)	9.3 [23]	15.8 [15]
	[75%–100%] (%, n)	3.7 [9]	17.9 [17]
	RV short axis diameter* (mm)	36.0 ± 7.0	38.0 ± 7.0
	LV short axis diameter* (mm)	44.0 ± 4.0	42.0 ± 6.0
	RV/LV diameter ratio	0.84 ± 0.19	0.91 ± 0.22

Definition of abbreviations: BMI, body mass index; SBP, systolic blood pressure; DBP, diastolic blood pressure; COPD, chronic obstructive pulmonary disease; CHD, coronary heart disease; CHF, congestive heart failure; WBCs, white blood cells; ALT, alanine aminotransferase; AST, aspartate aminotransferase; BUN, blood urea nitrogen; UA, uric acid; CRP, c-reactive protein; RBBB, right bundle branch block; TWI, T wave inversion; PA, pulmonary artery; RV, left ventricular; LV, right ventricular; NT-proBNP, N-terminal pro-B-type natriuretic peptide; CK-MB, creatine kinase and its MB isoenzyme; cTnT, cardiac troponin-T; cTnI, cardiac troponin I; MCTPA, multidetector computed tomography pulmonary angiography; ${\mathrm{SPO}}_{2}$, oxygen saturation. *detected by MCTPA.

In the derivation group, analysis of the ECG recordings on admission showed the presence of typical S1Q3T3 signs in 16 patients (6.5%), sinus tachycardia in 42 patients (17.1%), incomplete RBBB in 16 patients (6.5%), complete RBBB in 14 patients (5.7%), RAD in 6 patients (2.4%), atrial arrhythmias in 42 patients (17.1%) and V1–V3 TWI in 30 patients (12.2%). In the validation group, the most frequently observed abnormality was sinus tachycardia (n = 21, 22.1%), followed by incomplete atrial arrhythmias (n = 14, 14.7%), typical S1Q3T3 signs (n = 14, 14.7%), V1–V3 TWI (n = 13, 13.7%), incomplete RBBB (n = 8, 8.4%), RAD (n = 5, 5.3%) and complete RBBB (n = 2, 2.1%).

Among the 246 patients in the derivation group, 57 (23.2%) had thrombus in the left and/or right main PA, 59 (24%) in the lobar branches of PA and 118 (48.0%) in the segmental or subsegmental branches of PA. The mean Qanadli score in the derivation group was 27.5 (±25.3)%. In the validation group, MCTPA showed thrombus in the left and/or right main PA in 40 (42.1%) patients, in the lobar branches of PA in 25 (26.3%) patients and in the segmental or subsegmental branches of PA in 28 (29.5%) patients. The average Qanadli score in the validation group was 42.0 (±33.5)%.

### 3.3 Correlation between ECG Performance and Thrombus Burden 

**Fig. 3.**
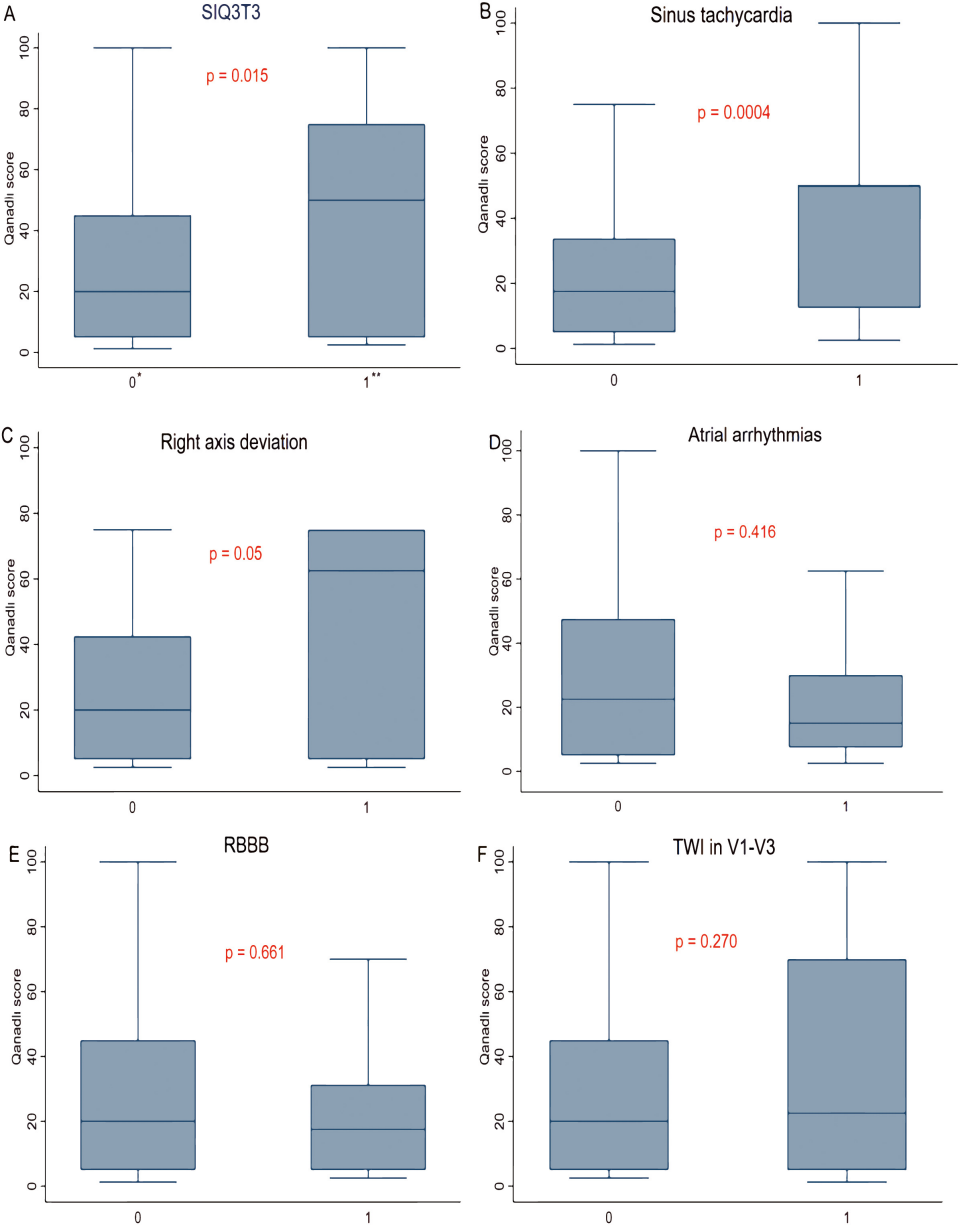
**Correlation between electrocardiogram abnormalities and Qanadli score.** (A) Association between the S1Q3T3 pattern and Qanadli score. (B) Association between sinus tachycardia and Qanadli score. (C) Association between right axis deviation and Qanadli score. (D) Association between atrial arrhythmias and Qanadli score. (E) Association between RBBB and Qanadli score. (F) Association between TWI in V1–V3 and Qanadli score. RBBB, right bundle branch block; TWI, T wave inversion. *absent = 0; **present = 1. *p* < 0.05 is referred as statistically significant and *p* < 0.001 as highly statistically significant.

The ECG abnormalities of S1Q3T3 signs (44.1% vs 26.9%, *p* = 0.015), sinus tachycardia (40.7% vs 25.0%, *p* = 0.004) and RAD (47.1% vs 26.8%, *p* = 0.05) were significantly associated with the Qanadli score. Although the Qanadli score was higher in patients with V1–V3 TWI (33.1% vs 27.2%, *p* = 0.270) on ECG, the difference was not statistically significant. The Qanadli score was lower in patients with either atrial arrhythmia (24.4% vs 28.2%, *p* = 0.416) or with RBBB (19.3% vs 29.4%, *p* = 0.661) on ECG, but neither of these differences was statistically significant (Fig. 3).

The distribution of ECG changes and thrombus sites are shown in Table 3. Univariable analyses revealed that acute PTE patients with S1Q3T3 signs (*p* < 0.01), sinus tachycardia (*p* < 0.01) or RAD (*p* < 0.05) on ECG were more likely to have a blood clot in the main PA. However, RBBB (*p* = 0.202), atrial arrhythmias (*p* = 0.218) and V1–V3 TWI (*p* = 0.406) were not significantly correlated with thrombosis location.

**Table 3.** **Correlation between electrocardiogram performance and location of thrombus**.

ECG changes	Location of thrombus			*p*
	Main PA	Lobar PA	Segmental PA	
S1Q3T3	7 [50.0%]	3 [21.4%]	4 [28.6%]	<0.001**
Sinus tachycardia	19 [46.3%]	10 [24.4%]	12 [29.3%]	<0.001**
RBBB	4 [13.3%]	6 [20.0%]	20 [66.7]	0.202
Right axis deviation	3 [50.0%]	0	3 [50.0%]	0.027*
Atrial arrhythmias	4 [10.5%]	12 [31.6%]	22 [57.9%]	0.218
TWI in V1–V3	9 [33.3%]	5 [18.5%]	13 [48.1%]	0.406

Definition of abbreviations: PA, pulmonary artery; RBBB, right bundle branch block; TWI, T wave inversion; ECG, electrocardiograph. * *p* < 0.05 is defined as statistically significant; ** *p* < 0.001 is defined as highly statistically significant.

### 3.4 Prognostic Model Development

**Fig. 4.**
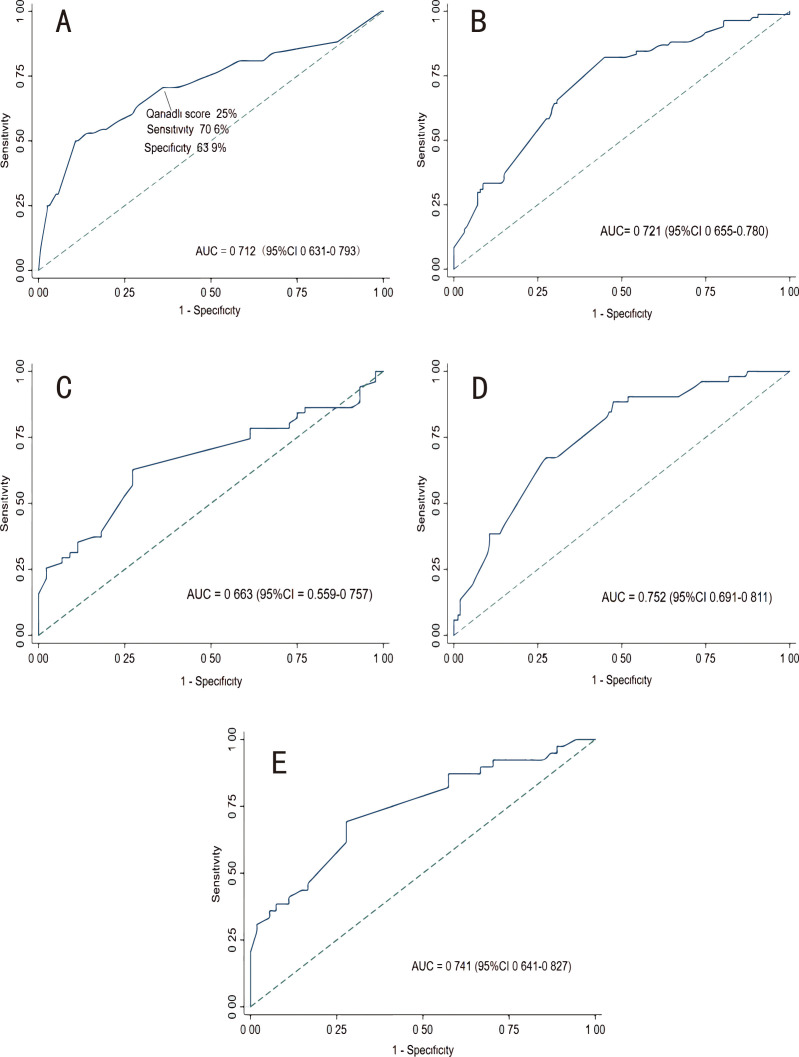
**Receiver operating characteristic curves.** (A) ROC curve for Qanadli score to predict right ventricular dysfunction. (B) ROC of CHARIS I model in the derivation cohort. (C) ROC of CHARIS I model in the external validation cohort. (D) ROC of CHARIS II model in the derivation cohort. (E) ROC of CHARIS II model in the external validation cohort. ROC, receiver operating characteristic curve; AUC, area under the curve; CHARIS, **C**OPD/CHF/CHD, **H**TN, **A**trial arrhythmias/AF, **R**BBB/RAD, **I**nverted T wave and **S**1Q3T3/ Sinus tachycardia.

The derivation dataset was used to create a ROC curve between Qanadli score and RVD detected by MCTPA. The best cut-off value obtained was 25% (70.6%, 63.9%) (Fig. 4A). To identify patients with a massive burden of thrombus, we used Qanadli score >25% as the dependent variable and then included our candidate predictors in a binary logistic regression model.

These new prediction models were given the acronym CHARIS (**C**OPD/CHF/CHD, **H**TN, **A**trial arrhythmias/AF, **R**BBB/RAD, **I**nverted T wave and **S**1Q3T3/ Sinus tachycardia).

The final risk equation, referred to here as the CHARIS I model (*p* < 0.01, $R^{2}$ = 0.109) was:

Probability of Qanadli score >25% = 1 / 1 + e^–risk score^. Risk score = 0.6 × S1Q3T3 signs + 1.0 × sinus tachycardia + 0.3 × RAD – 1.2 × RBBB – 0.5 × atrial arrhythmias + 0.5 × TWI in V1–V3 – 0.9 × COPD + 0.4 × HTN – 0.6 × CHD – 0.1 × CHF + 0.1 × AF – 0.473 (constant)

(Each factor in the equation is assigned a value of “0” if it is absent, and a value of “1” if it is present).

Similarly, we took thrombus in the main PA as the dependent variable and entered the same predictors as above into a binary logistic regression model to establish CHARIS II algorithm (*p* < 0.01, $R^{2}$ = 0.143):

Probability of thrombus in the main PA = 1 / 1 + e^–risk score^. Risk score = 0.6 × S1Q3T3 signs + 0.9 × sinus tachycardia + 1.1 × RAD – 0.9 × RBBB – 1.4 × atrial arrhythmias + 0.5 × TWI in V1–V3 – 1.0 × COPD + 0.3 × HTN – 0.6 × CHD – 1.1 × CHF – 0.2 × AF – 1.121 (constant)

(Each factor in the equation is assigned a value of “0” if it is absent, and a value of “1” if it is present).

### 3.5 Validation of the Prediction Models

To assess the performance of the CHARIS prognostication models, we applied the algorithms to both the derivation data set and the external validation data set.

#### 3.5.1 Discrimination

According to the ROC curves in Fig. 4, the CHARIS I model had good discriminative performance with an internally validated c-index of 0.721 (95% CI 0.655–0.780) and an externally validated c-index of 0.663 (95% CI 0.559–0.757). The AUC for CHARIS II model to distinguish thrombus in the main PA from thrombus in branch PA was 0.752 (95% CI 0.691–0.811) in the derivation set, while the c-index in the external validation set was 0.741 (95% CI 0.641–0.827).

#### 3.5.2 Calibration

**Fig. 5.**
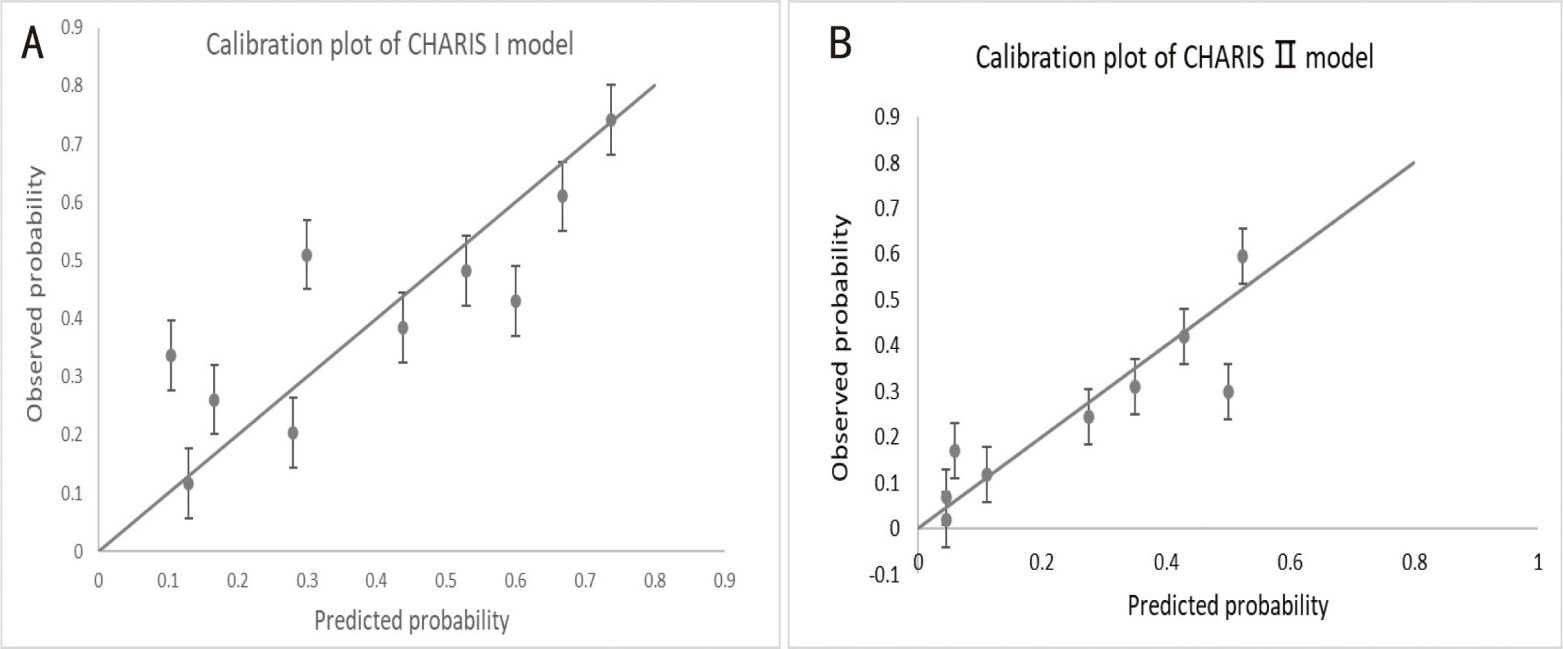
**Assessing calibration of the two models in the external validation cohort.** (A) Calibration plot of CHARIS I model with *p*-value for H-L test = 0.428 (*p* > 0.05), χ^2^ = 10.15. (B) Calibration plot of CHARIS Ⅱ model with *p*-value for H-L test = 0.927 (*p* > 0.05), χ^2^ = 3.76. The circles indicate the observed frequencies by decile of predicted probability. The solid line represents perfect calibration. CHARIS, **C**OPD/CHF/CHD, **H**TN, **A**trial arrhythmias/AF, **R**BBB/RAD, **I**nverted T wave and **S**1Q3T3/ Sinus tachycardia.

To further characterize the performance of accuracy, a calibration plot and the Hosmer-Lemeshow χ^2^ statistic for the two risk algorithms was performed in the external validation cohort. As evidenced by the generated calibration curves shown in Fig. 5, the agreement between the observed and predicted proportion of events indicated that both algorithms were well calibrated. The Hosmer-Lemeshow test results also corroborated the good calibration [CHARIS I model: χ^2^ = 10.15, *p* = 0.428 (*p* > 0.05); CHARIS II model: χ^2^ = 3.76, *p* = 0.927 (*p* > 0.05)].

#### 3.5.3 Clinical Utility

**Fig. 6.**
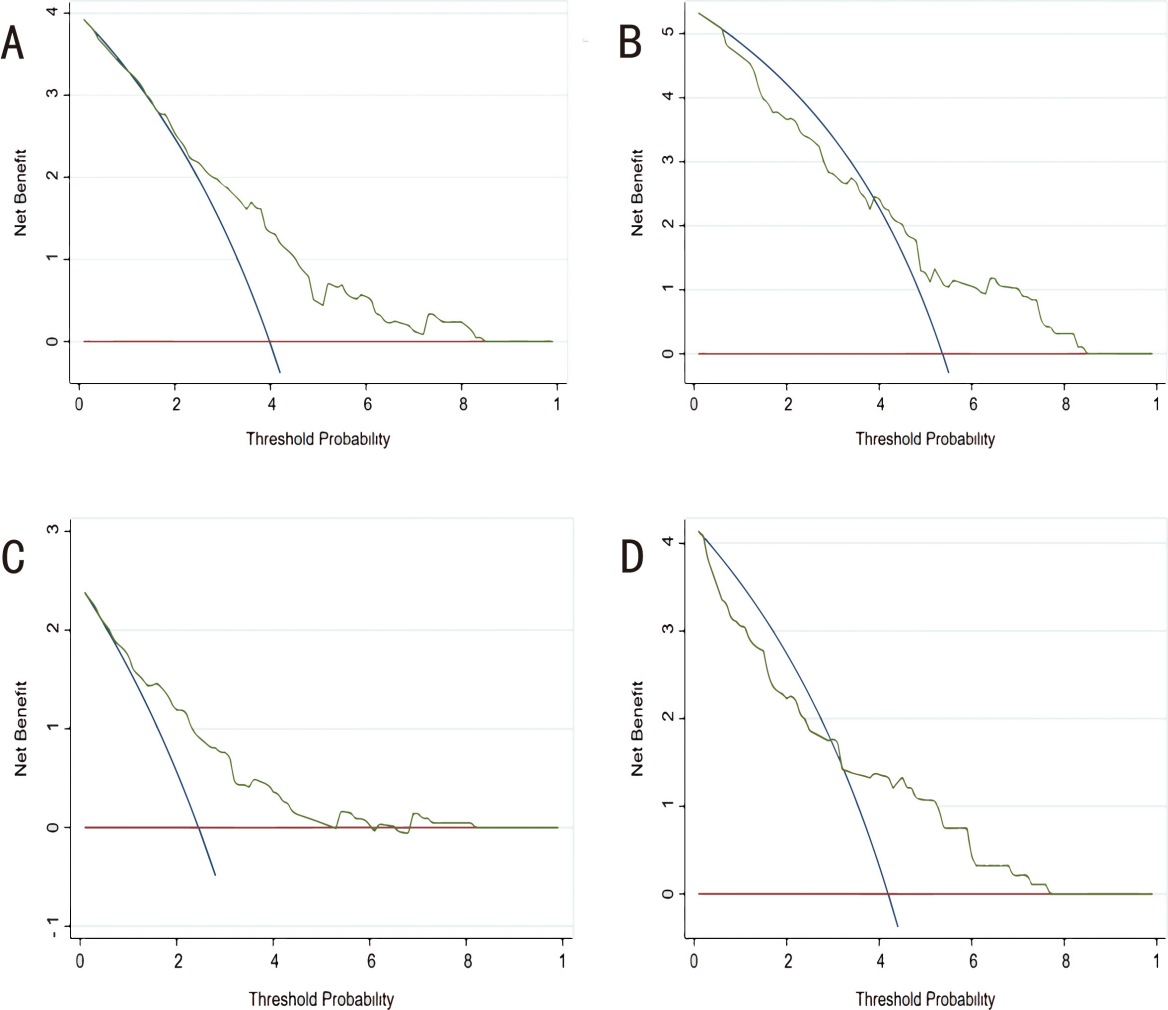
**Decision curve analysis (DCA) for each model in the derivation and validation cohorts.** (A) DCA of CHARIS I model in the derivation cohort. (B) DCA of CHARIS I model in the external validation cohort. (C) DCA of CHARIS Ⅱ model in the derivation cohort. (D) DCA of CHARIS II model for in the external validation cohort. CHARIS, **C**OPD/CHF/CHD, **H**TN, **A**trial arrhythmias/AF, **R**BBB/RAD, **I**nverted T wave and **S**1Q3T3/ Sinus tachycardia.

Finally, we carried out a decision curve analysis to evaluate the clinical utility of the novel models (Fig. 6). The CHARIS I model showed a positive net benefit across a broad range of risk thresholds from 20% to 90% in the derivation group and from 40% to 90% in the validation group. The CHARIS II model displayed consistent positive results and had a large net benefit at a threshold probability ranging from 10% to 90% in the derivation group and from 30% to 90% in the validation group.

## 4. Discussion

In the present study we found that the presence of the S1Q3T3 pattern, sinus tachycardia and RAD were associated with significantly heavier thrombotic load and were more frequent in patients with pulmonary trunk embolism than peripheral embolism. Furthermore, multivariate regression analysis revealed that sinus tachycardia was an independent predictor of thrombotic load (odds ratio (OR): 2.42; *p* = 0.016). In addition, we observed that RBBB, atrial arrhythmias and V1–V3 TWI showed trends for correlation with the Qanadli score or with the location of thrombus. There is mounting evidence to suggest these three ECG abnormalities are more frequent in cases with massive PTE than cases with sub-massive or no PTE, while still being valuable in the prognostic assessment of PTE [5, 6, 13, 14, 15, 16, 17, 18, 19, 20]. These paradoxical results may be due to our failure to screen out the new-onset RBBB, atrial arrhythmias and TWI in V1–V3, or because of the small sample size.

ECG changes in PTE essentially relates to dilatation of RV and alteration in the contractile properties of the RV myocardium induced by abrupt increase in pulmonary vascular resistance (PVR) [21]. Other pathophysiologic mechanisms that contribute to the ECG changes include PTE-induced release of inflammatory mediators, RV strain and hypoxia [22]. After reviewing 10 studies comprising 3007 patients with APTE, Shopp *et al*. [23] concluded that six findings of RV strain on 12-lead ECG (tachycardia, S1Q3T3, complete RBBB, TWI in V1–V4, ST elevation in aVR, and AF) were associated with an increased risk of circulatory shock and death.

Indeed, tachycardia is the only ECG abnormality included in both the original and simplified versions of the pulmonary embolism severity index (PESI) score [24, 25]. According to Kucher *et al*. [26], a heart rate >100 bpm without specifying the presence of sinus tachycardia or supraventricular tachycardia is correlated with an escalation of therapy [6]. Kukla *et al*. [20] showed that atrial fibrillation was present in 231 of 975 (24%) patients with APTE, and was associated with a higher risk of mortality (23% vs 12%, OR 2.1, *p* < 0.001) and complications (31% vs 20%, OR 1.8, *p* < 0.001). The S1Q3T3 pattern is one of the most typical ECG findings and is found more frequently in the setting of acute massive PTE [27]. Several studies have also found that RAD on ECG was associated with the severity of PTE and with a higher risk of adverse in-hospital courses [28, 29, 30], although others found no significant difference in this regard [6, 9, 14]. In addition, the frequency of RBBB in association with APTE has been reported to range from 6% to 69% [16, 31, 32, 33, 34, 35]. In the present study, RBBB was found in 12.2% of the derivation group and 10.5% of the validation group. Of note, PTE-related TWI is a repolarization abnormality that has been consistently reported as the most common ECG abnormality, with a variable frequency from 16% to 82.9% [17, 33, 36, 37, 38]. The PTE-related TWI frequency in the present study was 12.2%, which is lower than the frequencies reported in western countries. Although a uniform consensus has yet to be established for association of the above ECG abnormalities with adverse prognosis (including elevated cardiac biomarkers, RV enlargement, in-hospital complications or mortality), the potential utility of ECG findings as prognostic indicators for the severity of PTE is now widely recognized.

Rodrigues B, *et al*. [39] showed that a Qanadli score >18% was correlated with RV dysfunction on echocardiography (OR 10.85; 95% CI 3.20–36.7; *p* < 0.001). It is in line with the cut-off value obtained in the present study (25%), and lower than that obtained by Qanadli *et al*. [12] (40%) and Wu *et al*. [40] (60%). The result reflects differences in the populations studied.

To help distinguish patients with massive PTE from those with smaller PTE, we developed the CHARIS I and CHARIS II algorithms to calculate the risk of a Qanadli score >25% and the probability of trunk obstruction, respectively, in individual patients. Our models firstly combine the ECG findings with the comorbid diseases of COPD, HTN, CHD, CHF and AF. The risk prediction models undergo both internal and external validation processes. As expected, both algorithms were well suited for identifying patients in the derivation cohort with a heavy thrombus burden, as shown by a C-statistic of 0.72 and 0.75 for CHARIS I and CHARIS II, respectively. However, the prediction models showed slightly less discrimination in the external validation cohort. This could be interpreted by the imbalance of baseline characteristics between the two cohorts, based on the fact that patients in the derivation group had a lower mean Qanadli score (27.5% vs 42.0%, *p* < 0.001), while a greater proportion of patients in the validation group had trunk obstruction (42.1% vs 23.2%, *p* = 0.002). On the other hand, the calibration plot and Hosmer-Lemeshow test further confirmed and reinforced the accuracy of the prediction models in the external validation sample. Moreover, the DCA results suggest the rules were clinically useful, making them effective and feasible tools for the early identification of patients with a high Qanadli score or trunk obstruction prior to lung perfusion scintigraphy or pulmonary arteriography.

Currently, MCTPA is the preferred diagnostic modality for outpatients and emergency patients with suspected PTE. However, MCTPA is contraindicated for patients with history of severe contrast allergy, severe renal insufficiency, hyperthyroidism, or who are pregnant. Moreover, it is not available in remote areas and primary hospitals. 12-channel ECG is the most affordable, simple, secure, rapidly interpretable, repeatable and noninvasive test for the diagnosis and risk assessment of PTE. In summary, the present study used ECG signs and removed the impacts of several comorbidities on ECGs to develop two novel scoring systems that can help to estimate the clot burden of PTE and decisions regarding hospitalization.

## 5. Limitations

Several limitations of our study should be addressed. Firstly, despite being a multicenter study, the sample size of the derivation and validation cohorts was relatively small, and validation with a larger patient cohort is needed. Secondly, other ECG findings that were not included in our models, such as ST-segment depression (STD) [4, 6, 8, 10, 14], ST Elevation [6, 8, 10, 14, 41, 42], low QRS voltage in the peripheral leads [4, 8], QRS Fragmentation [8] and long QT [43]. These ECG changes were reported to provide valuable prognostic information in acute PTE. Therefore, the use of our rules to assist with diagnosis may result in some patients with massive PE being missed. Additionally, the severity of PTE in our study was defined by the degree of obstruction or filling defect on MCTPA. However, this is not in accordance with the current definition of massive PTE, which is characterized by hemodynamic collapse. Further research is needed to evaluate the accuracy of our algorithms for predicting the risk of adverse outcomes in patients with APTE. Another weakness of the study was that our models were not compared to previous scoring systems such as the Daniel score. Finally, patients with incomplete data were excluded on account of retrospective design, which may have introduced some patient selection bias. Despite these limitations, the greatest strength of the present investigation was the incorporation of several comorbid diseases into the models, thus reducing the confounding bias introduced by these comorbidities on the ECGs.

## 6. Conclusions

Algorithms were developed based on ECG abnormalities for quantitative and localization evaluation of thrombus burden in patients with APTE and then validated in an external population. The CHARIS I and CHARIS II models showed moderate discrimination and good calibration, while also demonstrating positive net benefits by decision curve analysis. Taken together, the novel algorithms were shown to be useful tools that could assist physicians when screening patients with massive PTE prior to imaging diagnosis, as well as in making decisions regarding patient hospitalization.

## Data Availability

The datasets used and analyzed during the current study are available from the corresponding author on reasonable request.
